# Bell’s palsy: a case report of unusual presentation in a patient with rhino-orbital cerebral mucormycosis

**DOI:** 10.1186/s13256-023-04298-x

**Published:** 2024-01-13

**Authors:** Elham Barahimi, Makiye Hamidi, Mehdi Hassani-Azad, Farzaneh Farshidi, Masoumeh Ardeshiri, MohammadHosein Sheybani-Arani

**Affiliations:** 1 Infectious and Tropical Diseases Research Center, Hormozgan University of Medical Sciences, Bandar Abbas, Iran; 2 Infectious and Tropical Diseases Research Center, Hormozgan Health Institute, Hormozgan University of Medical Sciences, Bandar Abbas, Iran; 3 Department of Pathology, Faculty of Medicine, Hormozgan University of Medical Sciences, Bandar Abbas, Iran; 4 Department of Radiology, Faculty of Medicine, Hormozgan University of Medical Sciences, Bandar Abbas, Iran; 5 Student Research Committee, Faculty of Medicine, Hormozgan University of Medical Sciences, Bandar Abbas, Iran

**Keywords:** Bell’s palsy, Fungal infection, Mucormycosis, Diabetes mellitus, Immunocompromised patient

## Abstract

**Background:**

Mucormycosis is a fungal infection caused by the Mucorales order of fungi. This fungus is commonly found in soil and can cause disease in immunocompromised patients. On the other hand, Bell’s palsy is an idiopathic condition that results in the sudden onset of unilateral facial muscle weakness, affecting the facial nerve.

**Case presentation:**

A 51-year-old Persian housewife with a history of poorly controlled diabetes mellitus presented with a splitting headache that had been ongoing for 1 week and an inability to close her left eye or make facial expressions on the left side of her face. The patient’s vital signs were normal, but physical examination revealed a yellow-grey scar on the left side of her hard palate and Bell’s palsy on the left side. A neurological examination showed that she could move both eyes but could not close her left eye, move up her left eyebrow, or smile. Further investigations were performed, including laboratory tests, radiologic imaging, and functional endoscopic sinus surgery. The patient underwent three rounds of debridement for bony erosion in the medial and posterior walls of the left maxillary sinus and the hard palate. Pathological examination confirmed mucormycosis infection in the hard palate and mucosa.

**Conclusion:**

Fungal infection must be considered a potential diagnosis for immunocompromised adults who exhibit symptoms of Bell’s palsy.

## Introduction

Mucormycosis is a fungal air-borne infection caused by a group of opportunistic moulds known as mucormycetes, which enter the respiratory tract via inhalation, pass through it, and colonise any part of it [1–3]. Rhizopus, Apophysomyces, Mucor, and Lichtheimia are its species with global distribution [4]. Nasal turbinates are known sites for this colonisation, and based on their anatomical proximity to orbit, sinuses, and cerebrum, the infection can show diverse presentations [5].

Bell's palsy is the most common cranial nerve disease, an idiopathic form of facial nerve palsy [6]. The sudden onset of unilateral facial muscle weakness is a cardinal sign of Bell's palsy that can be accompanied by drooling, tenderness behind the ear, ipsilateral cheek paraesthesia, and alteration in taste and loudness perception [7, 8].

## Case presentation

**Fig. 1:**
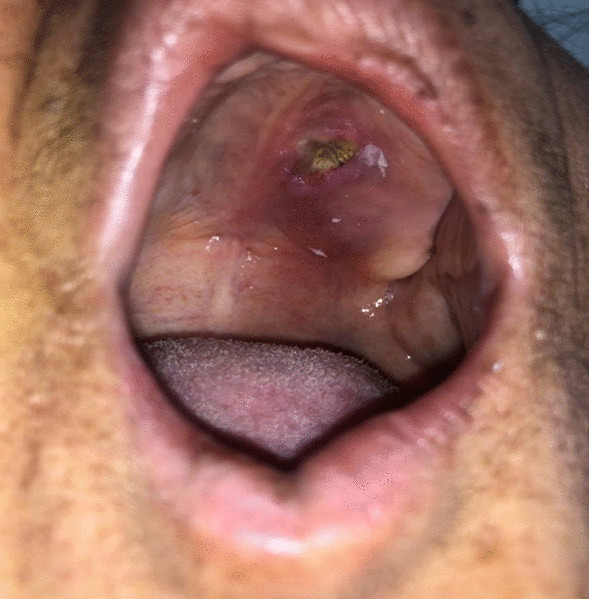
A yellow-grey eschar 1*1 cm (cm) in diameter was noted on the left side of the hard palate

A 51-year-old housewife Iranian woman from Hormozgan province in southern Iran was admitted to the hospital with a history of poorly controlled diabetes Mellitus and chief complaints of constant splitting headaches in the left frontal area that had been going on for one week. She had no nausea or vomiting, but she was sensitive to light and could not close or wink her left eye, smile, make a unilateral facial deviation, or raise her left brow. The patient had a past medical history of Type II diabetes mellitus and was taking metformin and gliclazide with no history of drug allergies. She was ill in general appearance. Vital signs were within the normal range, and during the physical examination, a yellow-grey eschar 1*1 cm (cm) in diameter was noted on the left side of the hard palate (Fig. 1). In the neurological examination, she could move both eyes but could not close her left eye, move up her left eyebrow, or smile. The rest of the systemic examination did not reveal abnormalities. According to the patient history and physical examination, mucormycosis was among the differential diagnoses considered.

Laboratory tests revealed a marked increase in white blood cells (WBC), estimated sedimentation rate (ESR), C-reactive protein (CRP), Hemoglobin subunit alpha (HbA1C), and decreased haemoglobin (HB). Other findings were unremarkable (Table 1).

**Table 1:** Laboratory data of case

Lab data	Result	Reference range
WBC (10^9^/L)	14.7	4–11
Hb (g/dL)	9.5	13–16
MCV(Fl)	87	80–100
PLT(10^3^/μL)	655	150–450
Creatinine (mg/dL)	0.8	0.6–1.3
Urea (mg/dL)	35	11–55
LDH (U/L)	682	120–460
SGOT (U/L)	14	< 37
SGPT (U/L)	21	< 41
Alp (U/L)	309	100–360
CRP (mg/dL)	38.6	0–6
Na (mg/dL)	134	135–145
K (mg/dL)	3.8	3.6–5
ESR(mm/h)	68	0–15
HIV-P24	0.10	Negative: < 0.25 Positive: 0.25

*WBC* White Blood Cells; *Hb* Hemoglobin; *MCV* Mean Corpuscular Volume; *PLT* Platelet Count; *LDH* Lactate Dehydrogenase; *SGOT* Serum Glutamic Oxaloacetic Transaminase; *SGPT* Serum Glutamic-Pyruvic Transaminase; *Alp* Alkaline Phosphatase; *CRP* C-Reactive Protein; *Na* Sodium; *K* Potassium; *ESR* Erythrocyte Sedimentation Rate; *HIV-P24* Human Immunodeficiency Virus P24 (antigen)

**Fig. 2:**
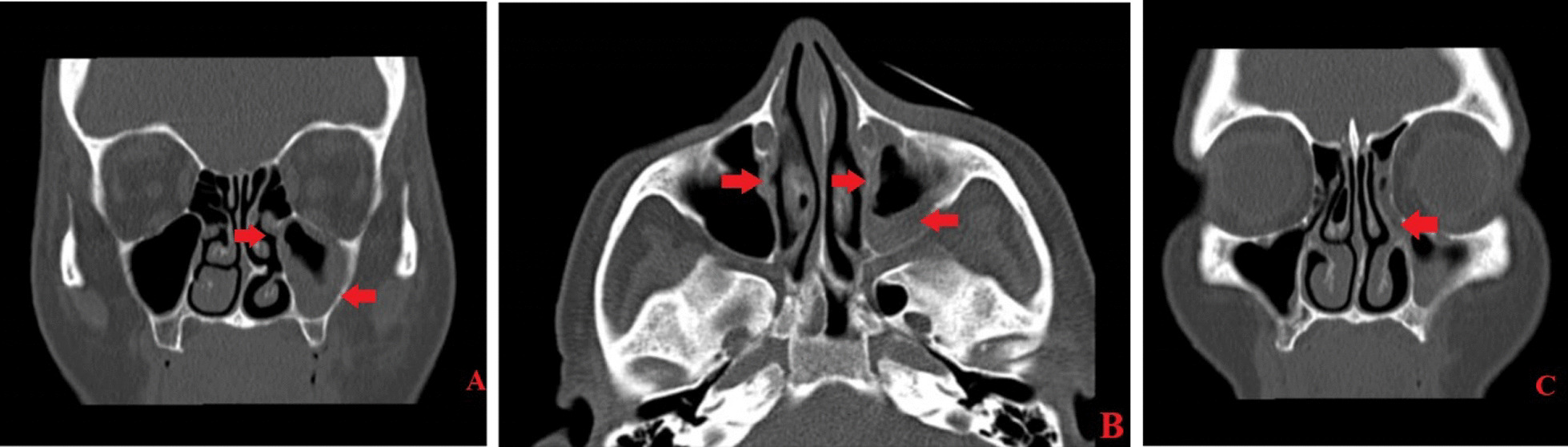
In the **A** (coronal image) and **B** (Axial image), evidence of near total opacification of the left maxillary sinus (right arrow, parts **A** and **B**) is seen that extends into the left ethmoidal air cells. Also, bone remodelling in the left ethmoidal sinus and posterior, superior and medial walls of the left Maxillary sinus is noted (middle arrow, part **B** and left arrow, part **A**). Mild mucosal thickening of the right maxillary sinus (left arrow, part **B**) and inferior nasal concha are seen, too. In **C** (coronal image), opacification and widening of the left side Osteomeatal complex are seen (right arrow, part **C**)

**Fig. 3:**
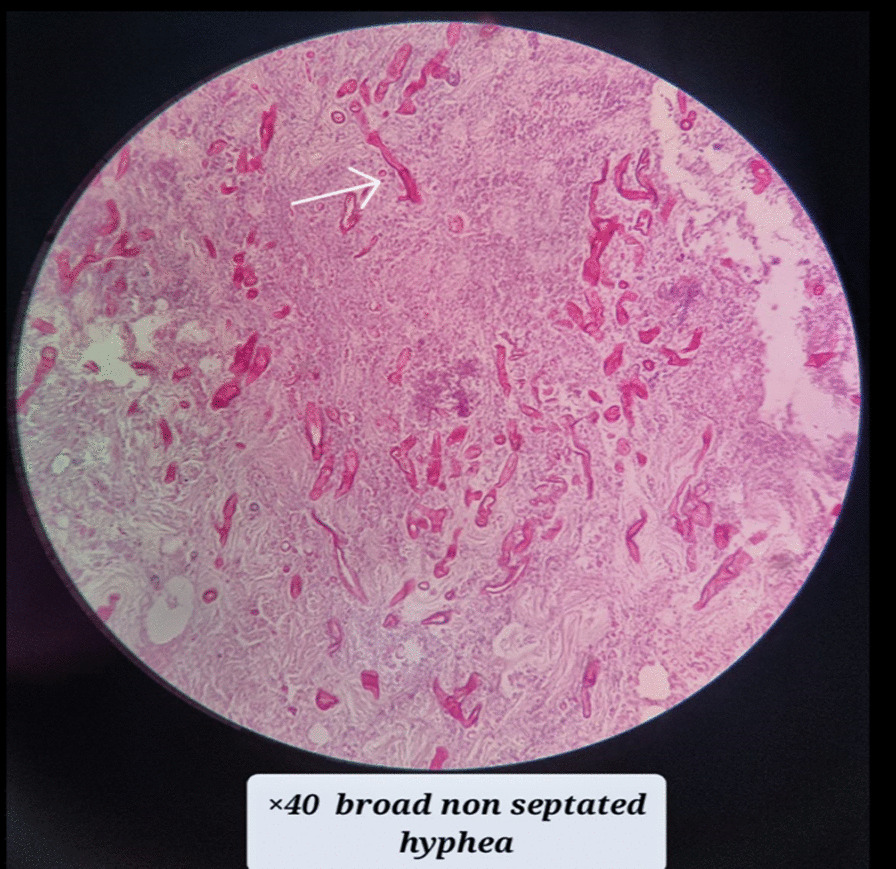
Necrotic tissue with numerous broad nonseptate ribbon-like fungal hyphae (arrow), indicating mucormycosis infection

A spiral paranasal sinus Computed Tomography (CT) scan without contrast revealed near-total opacification of the left maxillary sinus, left nasal cavity and left ethmoidal sinus. Mucosal thickening was observed in almost all paranasal sinuses, and bony erosion was noted in the medial and posterior walls of the left maxillary sinus (Fig. 2). Following the oral lesion and paranasal sinus CT scan, amphotericin-B treatment was initiated, and an ear, nose and throat (ENT) consultation was scheduled. The patient underwent immediate functional endoscopic sinus surgery as a result. Biopsy results of the hard palate and mucosa indicated necrotic tissue with numerous broad nonseptate ribbon-like fungal hyphae, indicating mucormycosis infection (Fig. 3). To treat this condition, the patient continued treatment with amphotericin B deoxycholate at a dosage of 1 mg/kg/day for a month and underwent three rounds of debridement.

Due to the persistence of her headache, the patient underwent an extensive diagnostic workup, which included brain Magnetic Resonance Imaging (MRI) without contrast, Brain Magnetic Resonance Venography (MRV), and brain Magnetic Resonance Angiography (MRA). Additionally, her headache, left eye discomfort, and facial movement improved after 1 month of hospital treatment. The patient was discharged with a prescription for posaconazole 200 mg every 6 h and gabapentin 100 mg every night for three months and starting insulin therapy. A follow-up visit three months later revealed successful treatment of the infection.

## Discussion

Bell’s palsy is a condition that can affect individuals of all ages and genders, causing facial paresis or paralysis. This can result in temporary oral insufficiency and, in some cases, an inability to close the eyelids. This disease usually causes facial asymmetry [9]. Mucormycosis is an infection caused by Mucorales, found in soil, bread, and dust. This infection can occur through inhaling spores into the respiratory tract, ingesting contaminated food, or inoculating disrupted skin. Mucormycosis is an opportunistic and rare but aggressive fungal infection that causes diseases in immunocompromised patients [9, 10]. This case presents with a splitting headache, facial palsy, inability to close her left eyelid or wink, make a smile unilateral facial deviation, or move her left eyebrow up. On physical examination, the left side of the hard palate has yellow-grey eschar and has a risk factor, including uncontrolled diabetes mellitus.

The mortality rate associated with mucormycosis is alarmingly high, with some case series reporting rates as high as 80%. Therefore, a prompt diagnosis must be made as treatment initiation is time-sensitive due to the rapid progression of the infection. Diagnosis typically involves histopathology and tissue culture. These fungi can cause a range of clinical syndromes, with specific manifestations depending on the side of the infection. Seven different syndromes may occur, including the rhino-orbital cerebral, pulmonary, gastrointestinal, central nervous system, cutaneous, miscellaneous, and disseminated. The recommended treatment for mucormycosis involves administering amphotericin B Liposomal type. However, it is often prohibitively expensive or unavailable in many resource-limited settings. Following this, the treatment can be continued with posaconazole, which has been shown to have a response rate of 60%-70%. For effective treatment, rhino-cerebral mucormycosis requires extensive debridement and adequate amphotericin B [4, 11–14].

Mucormycosis infection, in this case, was proved by the biopsy results of the hard palate and mucosa that indicated necrotic tissue with numerous broad nonseptate ribbon-like fungal hyphae. Upon observing the yellow-grey oral lesion and paranasal sinus CT scan, treatment with amphotericin B and debridement was promptly initiated.

## Conclusion

In conclusion, an unusual instance of facial Bell’s palsy caused by mucormycosis in an immunocompromised individual has been reported. The presence of sinus pain, congestion, headache, necrotic and exophytic lesions in the hard palate, facial nerve palsy with involvement of the sinuses in the CT scan in the form of opacification, increased mucosal thickness, air-fluid level and bone erosion in immunosuppressed individuals, clinical suspicion of mucormycosis should be considered. Healthcare professionals need to recognise these distinctive clinical symptoms, as early intervention with appropriate medical and surgical treatment can improve disease recovery and lead to a better prognosis with minimal complications.

## Data Availability

The data sets used during the current study are available from the corresponding author upon reasonable request.

## Abbreviations

Cm: Centimetre
WBC: White blood cells
ESR: Estimated sedimentation rate
CRP: C-reactive protein
HbA1C: Haemoglobin subunit alpha
HB: Haemoglobin
CT: Computed Tomography
ENT: Ear, Nose, and Throat
MRI: Magnetic Resonance Imaging
MRV: Magnetic Resonance Venography
MRA: Magnetic Resonance Angiography
